# Determinants of the daily rhythm of blood fluidity

**DOI:** 10.1186/1740-3391-7-7

**Published:** 2009-06-26

**Authors:** Tatsushi Kimura, Tsutomu Inamizu, Kiyokazu Sekikawa, Masayuki Kakehashi, Kiyoshi Onari

**Affiliations:** 1 Yasuda Women's College, Department of Kindergarten Education, Yasuhigashi 6-13-1, Asaminami-ku, Hiroshima 731-0153, Japan; 2 Hiroshima University, Graduate School of Health Science, Institute of Health Sciences, Kasumi 1-2-3, Minami-ku, Hiroshima 734-0037, Japan; 3 Fukuyama Heisei University, Department of Health and Sport Sciences, Miyuki Kamiiwanari 117-1, Fukuyama, Hiroshima 720-0001, Japan

## Abstract

**Background:**

Numerous processes in the living body exhibit daily rhythmicity. In this study, we characterized a daily rhythm of blood fluidity and identified its determinants.

**Methods:**

The subjects were nine young males. We measured the physiological parameters and performed hematological and biochemical analyses. We repeated the measurements six times during the day at 7:30 (just after getting up and before breakfast), 10:00, 13:30 (after lunch), 16:30, 19:30 (after dinner), and 21:30. The subjects performed sedentary work all day, and the contents and time of the meals were uniform. Investigation of blood rheology was based on Kikuchi's microchannel method.

**Results:**

Blood passage time varied significantly with time of day. Stepwise regression analysis was used to determine the significant factors affecting blood passage time. Body temperature, heartbeat, hematocrit, white blood cell and total cholesterol were significant determinants of blood passage time.

**Conclusion:**

We confirmed that blood fluidity has a daily rhythm. In addition, we found that the determinants of blood fluidity included physiological parameters such as body temperature and heartbeat, hematological parameters such as hematocrit, and white blood cell and total cholesterol.

## Background

Research conducted during the past half century has made it clear that virtually all physiological phenomena in the living body exhibit daily rhythmicity. Refinetti and Menaker [1] reviewed the literature on the circadian rhythm of body temperature, emphasizing species differences, development and aging, and the relationships between the circadian rhythm of body temperature and the circadian rhythm of daily activity. In the clinical field, previous studies [2-4] have documented an important role of circadian rhythmicity in cardiovascular health. Uehara et al [5] reported that platelet aggregation and blood coagulation have a time course change and that these may have implications for cerebral infarction. On the other hand, in the sports field, it is common for athletes to adjust the hour of rising and timing of warming up according to the start of a game. It is necessary to adjust the hour of rising to achieve the best performance based on body temperature and heartbeat. In the present study, we characterized a daily rhythm of blood fluidity and identified its determinants.

## Methods

### Subject

The subjects were nine clinically healthy men who were non-athletes and non-smokers (age: 22.6 ± 1.7 yr, height: 171.1 ± 7.5 cm, weight: 68.5 ± 12.7 kg). They gave consent to participate after an explanation of the study and the measurements needed. The study was approved by the Ethics Committee of Hiroshima University, Graduate School of Health Science, Department of Physical Therapy and Occupational Therapy Sciences (No.0554).

### Protocol

We asked the subjects to avoid overeating, overdrinking and indulging in high intensity exercise on the night before the experiment, asked them to remain seated all day in a laboratory, and asked them to avoid exercise other than necessary everyday movement on the experiment day. The subjects ate three meals of the same contents at standard times. The drinks between meals were only mineral water, and snacks were consolidated as much as possible. The subjects moved to a laboratory immediately after getting up from bed.

We separated the subjects into three groups and conducted measurements at the same time for the three groups. The measurements were taken six times: at 7:30 (just after getting up, before breakfast), 10:00, 13:30 (after lunch), 16:30, 19:30 (after dinner), and 21:30.

### Measured items

The measured items were body temperature, heartbeat, blood pressure, whole blood passage time, a hematological parameter and a biochemical parameter. Blood samples were drawn from subjects in a seated position from an antecubital vein with anticoagulation by heparin solution (1000 IU/ml; 0.5 parts to 9.5 parts blood) after resting in a chair for at least five minutes. The volume of the blood sample was 10 ml (5 ml × 2 vacuum tubes). To avoid repeated puncture, the second tube was used for the measurement of blood passage time. The measurement of blood passage time was done as soon as possible after blood samples were drawn. A mercury thermometer was used for 15 minutes to measure axillary body temperature. We used an automatic sphygmomanometer (Omron Co., Ltd.; HEM-757) to measure blood pressure and heartbeat. They were measured three times consecutively and the mean of the three measurements was used. We placed the cuff (manchette) of the sphygmomanometer precisely at a unified position and height. An automatic cell counter (Beckman Coulter Co. Ltd.) was used to measure red blood cell, white blood cell, hemoglobin, hematocrit, and platelet. The clinical laboratory at the hospital conducted analysis of albumin (Bromocresyl green method), fibrinogen (Laser light scattering method), total protein (Biuret method), beta-lipo protein (Turbidimetric Immuno Assay method), total cholesterol (Enzyme method), HDL-cholesterol (Homogeneous method), tri-glyceride (Glycerin-1-phoshate-oxydase method), and blood glucose (Hexokinase glucose-6-pyruvate dehydrogenase method).

### Determination of blood rheology

Investigation of blood rheology was based on Kikuchi's microchannel method [6-8]. Microgrooves (width 7 μm, length 30 μm, depth 4.5 μm) were photo-fabricated on the surface of a single crystal silicon substrate (chip dimensions 15 × 15 mm). We converted microgrooves into leak-proof microchannels by tightly covering them with an optically flat glass plate. The groove was transformed into a hermetic microchannel by soldering it to an optically polished glass plate. Because the volume of fluid which flows through one flow path is extremely small, 8736 flow paths of the same size were created to make it possible for measuring the flow rate. The silicon single crystal substrate was then mounted onto the microchannel flow system, MC-FAN (Hitachi Haramachi Electronics Co., Ltd, Ibaragi, Japan). This system makes it possible to directly observe the flow of blood cell elements through the microchannel under a microscope connected to an image display unit. In this system, flow can be continuously viewed while the passage time for a given volume of blood is determined automatically. Our revised value of blood passage was expressed as a function of the actual whole blood passage time over saline solution passage time of 12 seconds at a pressure of 20 cm H_2_O.



### Statistics

We used Stat View 5.0 (SAS) to calculate statistics and SPSS 12.0 (SPSS) to do multiple regression analysis (forward selection). We used one-way repeated-measures ANOVA and used Fisher's PLSD in the case that a significant difference was recognized. We used multiple regression analysis to analyze factors that determine the daily rhythm of blood fluidity. Differences associate with P < 0.05 were considered statistically significant.

## Results

### Physiological parameters (Table 1, Fig. 1)

**Table 1:** Time-course of physical and hematological parameters

		7:30	10:00	13:30	16:30	19:30	21:30	P value
Body temperature	(°C)	36.5 ± 0.1	36.7 ± 0.2	36.9 ± 0.2	36.7 ± 0.2	36.9 ± 0.2	36.9 ± 0.2	< 0.001
Heartbeat	(beat/min)	61.0 ± 5.7	66.8 ± 8.0	66.3 ± 5.0	63.6 ± 6.1	68.0 ± 6.2	67.4 ± 7.5	< 0.01
Systolic blood pressure	(hPa)	144.9 ± 12.8	147.9 ± 13.2	152.2 ± 14.6	145.4 ± 9.3	149.4 ± 11.6	153.5 ± 14.3	< 0.05
Diastolic blood pressure	(hPa)	92.4 ± 8.6	84.4 ± 9.0	81.0 ± 7.0	89.2 ± 12.0	89.1 ± 9.8	89.4 ± 11.9	< 0.01
Blood passage time	(second)	50.5 ± 3.5	46.8 ± 3.1	45.4 ± 2.5	48.5 ± 3.2	46.6 ± 2.6	46.5 ± 2.1	< 0.001
Hematocrit	(%)	46.7 ± 2.2	46.5 ± 2.4	45.4 ± 1.7	46.9 ± 1.7	45.8 ± 1.6	45.6 ± 1.9	< 0.01
Red blood cell	(× 10^4^cells/μl)	487.4 ± 30.7	487.4 ± 31.4	474.4 ± 27.3	490.8 ± 25.1	478.6 ± 30.2	471.0 ± 29.1	< 0.05
Hemoglobin	(mg/dl)	14.9 ± 0.7	14.8 ± 0.8	14.4 ± 0.6	14.9 ± 0.7	14.5 ± 0.6	14.2 ± 0.6	< 0.01
White blood cell	(cells/μl)	5511.1 ± 1267.3	5200.0 ± 1136.9	5388.9 ± 869.5	5600.0 ± 933.5	5933.3 ± 919.2	6350.0 ± 750.3	0.06
Platelet	(× 10^4^cells/μl)	21.6 ± 4.4	22.0 ± 4.5	21.6 ± 3.9	22.3 ± 3.5	21.3 ± 3.4	21.9 ± 4.7	0.83
Albuminn	(g/dl)	4.7 ± 0.2	4.6 ± 0.1	4.7 ± 0.2	4.7 ± 0.2	4.7 ± 0.2	4.7 ± 0.1	0.27
Fibrinogen	(mg/dl)	201.9 ± 20.4	200.8 ± 24.5	193.3 ± 22.4	196.9 ± 22.8	192.4 ± 26.0	198.9 ± 30.7	0.09
Total cholesterol	(mg/dl)	166.1 ± 16.9	165.2 ± 14.5	162.6 ± 12.4	164.2 ± 13.1	159.6 ± 12.9	160.6 ± 13.6	< 0.01
HDL-cholesterol	(mg/dl)	58.2 ± 9.4	55.4 ± 8.9	54.9 ± 9.5	59.1 ± 8.6	57.8 ± 9.6	60.0 ± 9.1	< 0.001
Total protein	(g/dl)	7.3 ± 0.3	7.3 ± 0.2	7.3 ± 0.3	7.4 ± 0.3	7.3 ± 0.3	7.2 ± 0.2	0.18
Beta-lipo protein	(mg/dl)	259.7 ± 51.8	288.9 ± 59.3	281.0 ± 59.8	248.9 ± 47.4	255.4 ± 40.5	233.8 ± 37.3	< 0.001
Tri-glyceride	(mg/dl)	69.2 ± 45.9	151.7 ± 88.2	131.0 ± 75.7	63.4 ± 31.1	111.6 ± 48.7	46.0 ± 16.7	< 0.001
Blood glucose	(mg/dl)	82.6 ± 3.0	74.2 ± 10.4	85.8 ± 14.5	84.6 ± 7.7	96.9 ± 18.9	98.2 ± 16.4	< 0.001

Values shown are means ± standard deviations for nine subjects.

P value shows the result of ANOVA of one way repeated measures (Fisher's PLSD) and the value smaller than 0.05 shows significant change for the time course.

**Figure 1:**
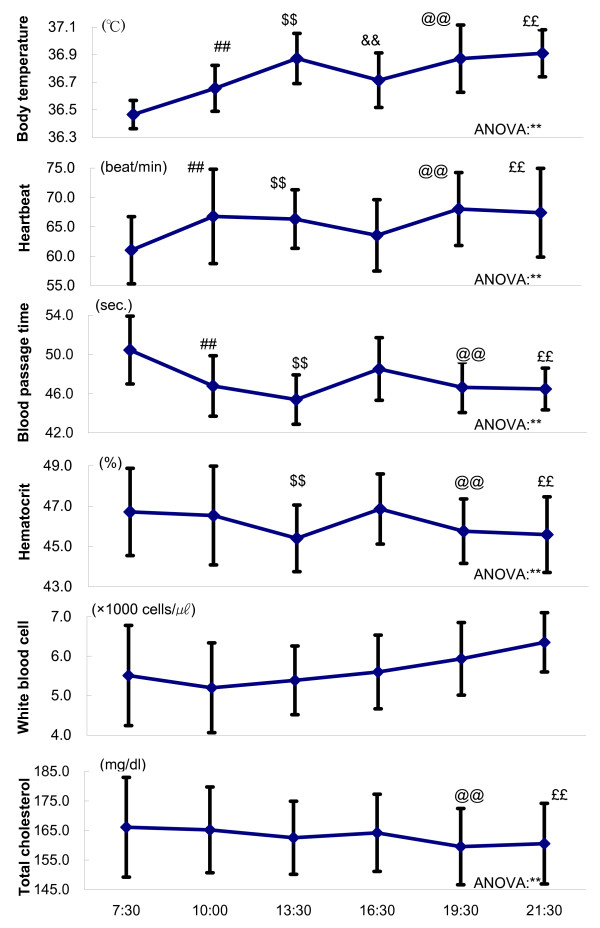
**Time-course of physiological and hematological parameters**. Each point shows mean ± standard deviations. ANOVA suggests a robust daily rhythm of body temperature, heartbeat, blood passage time, hematocrit and total cholesterol. ** P < 0.01 Fisher's PLSD: # P < 0.05, ## P < 0.01; 7:30 v.s.10:30, $ P < 0.05, $$ P < 0.01;7:30v.s.13:30, & P < 0.05, && P < 0.01;7:30v.s.16:30, @ P < 0.05, @@ P < 0.01;7:30v.s.19:30, £ P < 0.05, ££ P < 0.01;7:30v.s.21:30

All of the physiological parameters showed significant change with time of day, some of them rising through the day, others falling through the day. Blood passage time and body temperature seemed to be negatively correlated (R^2 ^= 0.826), whereas blood passage time and diastolic blood pressure seemed to be positively correlated (R^2 ^= 0.593).

### Hematological parameters (Table 1, Fig. 1) 

 Blood passage time, hematocrit, red blood cell and hemoglobin showed significant change with time of day. There were positive correlations between blood passage time and hematocrit (R^2 ^= 0.643), between blood passage time and red blood cell (R^2 ^= 0.480), and between blood passage time and hemoglobin (R^2 ^= 0.551). White blood cell, platelet, albumin and fibrinogen did not show significant changes with time of day.

### Blood biochemical parameters (Table 1, Fig. 1)

Total cholesterol, HDL-cholesterol, beta-lipo protein, tri-glyceride and blood glucose showed significant change with time of day. These parameters fluctuated through the day. There were no significant correlations between blood passage time and blood biochemical parameters (P > 0.10).

### Multiple regression analysis (Table 2)

**Table 2:** Stepwise regression analysis

Variables	B	SEB	β	r	R^2^	SEE
Step 1						
Hematocrit	0.857	0.205	0.506	0.506	0.257	2.74

Step 2						
Hematocrit	1.117	0.182	0.66	0.691	0.478	2.32
Heartbeat	-0.233	0.051	-0.495			

Step 3						
Hematocrit	1.063	0.171	0.627	0.744	0.553	2.17
Heartbeat	-0.178	0.051	-0.379			
White blood cell	0.925	0.321	0.296			

Step 4						
Hematocrit	0.843	0.169	0.497	0.799	0.638	1.97
Heartbeat	-0.047	0.061	-0.099			
White blood cell	1.399	0.325	0.448			
Body temperature	-5.223	1.558	-0.388			

Step 5						
Hematocrit	1.207	0.239	0.713	0.818	0.669	1.91
Heartbeat	-0.023	0.06	-0.048			
White blood cell	1.722	0.35	0.551			
Body temperature	-5.414	1.509	-0.402			
Total cholesterol	-0.07	0.034	-0.305			

B:standardised partial regression coefficient, SEB:standard error of regression coefficient,

β: normal partial regression coefficient, r:coefficient of correlation,

R^2^:decision coefficient,

SEE:standard error of the estimate

Stepwise regression analysis was used to determine the significant factors which affect largely the blood passage time. As a result of analysis, we have found that body temperature, heartbeat, hematocrit, white blood cell and total cholesterol were significant and then we could explain 66.9% of the whole blood passage time by these five parameters.

The prediction formula is as follows;



## Discussion

Blood passage time showed significant change with time of day, suggesting the existence of a daily rhythm of blood fluidity. The maximum value for blood passage time was after getting up in the morning (before breakfast). At that time, blood fluidity was at its worst for the day. Then, blood passage time fell gradually after breakfast and again after lunch. The value at 13:30 was the minimum. At that time, blood fluidity was the best of the day. Okazaki et al [9] reported that endurance exercise training shortened whole blood passage time with the increase in exercise amount of training by decreasing red cell count and hematocrit. Moderate exercise shortened whole blood passage time and brought good physical performance, thus indicating that short blood passage time is advantageous to the oxygen transport.

We used multiple regression analysis to try to identify the factors that determine the daily rhythm of blood fluidity. Based on multiple regression analysis, we inferred that body temperature and heartbeat may be determinants of the daily rhythm of blood fluidity. Body temperature showed an especially strong correlation with blood passage time, with its change over the day being diametrically opposite to that of blood passage time. It is conceivable that this effect derives from a general temperature dependence of the fluidity in viscous material. Multiple regression analysis also suggested that hematocrit and white blood cell count are determinants of the daily rhythm of blood fluidity. We confirmed that hematocrit affected blood passage time as was reported in a previous study [10]. The biochemical parameters of total cholesterol, HDL-cholesterol, beta-lipo protein, tri-glyceride and blood glucose showed significant change with time of day, while albumin and total protein did not. Albumin is an important protein regarding blood fluidity. Takahashi et al [11] reported that albumin had a negative correlation with whole blood passage, but the measured values of albumin were at a normal level. So, they concluded that the relevance of plasma lipid concentration for blood fluidity was weak. On the other hand, another study [12] reported that in athletes the correlations of red cell aggregation with plasma fibrinogen weakened in both young and old red blood cell populations while albumin became more significant. In this study, albumin did not show significant change with time of day, and the measured vales were at a normal level. Significant correlation between LDL-cholesterol in plasma and membrane cholesterol was observed. We believe that mixed hyperlipidemia may have influenced the erythrocyte membrane structure, which caused significant decrease of membrane fluidity in the superficial layer without any significant changes in deeper layer and significant increase of membrane cholesterol and thiobarbituric acid reaction substances. HDL-cholesterol, beta-lipo protein, tri-glyceride and blood glucose did not show a strong correlation to blood passage time. We consider total cholesterol to be a determinant of the daily rhythm of blood fluidity. Hunter et al [13] investigated the relationship of the effects of three isoenergic diets of differing fat composition and the variables of blood coagulation. They could not find significant relationship in platelet aggregation response and membrane fluidity observed in any of the diets. Shimabukuro et al [14] reported that triacylglycerol, insulin, glucose, total cholesterol, HDL-cholesterol and adiponectin were not correlated with decreases in peak forearm blood flow and flow debt repayment after a high-fat meal. We have not found the reason why total cholesterol affects blood fluidity. Another study [15] showed that, in the group of patients with mixed hyperlipidemia, there was a significant correlation between LDL-cholesterol in plasma and membrane cholesterol. We think that cholesterol may have some influence on the cell membrane.

In the case of sports and exercise for health, adjusting the time of a game or exercise to when whole blood passage time is minimum (advantage to the oxygen transport) should be important, as it may induce one's best performance. According to our results, people had better avoid doing exercise when whole blood passage time is at a high value (oxygen may not be delivered smoothly to the tissue), i.e. early in the morning. Further studies are needed to solidify this inference. Also, we must evaluate changes in blood passage time before and after exercise bouts and along the sleep-wake cycle [16,17] before we can recommend the best time of day for exercise and eating.

## Conclusion

We confirmed that there is a daily rhythm in blood fluidity. Because blood passage time was the longest at 7:30 (just after getting up and before breakfast) and the shortest 13:30, we estimate that the best time of day for safe and effective exercise is the early afternoon and, conversely, that exercise should be avoided early in the morning.

Multiple regression analysis suggested that physiological parameters such as body temperature and heartbeat, hematological parameters such as hematocrit and white blood cell count, and biochemical parameters such as total cholesterol were factors determining the rhythmic pattern of blood fluidity.

## Competing interests

The authors declare that they have no competing interests.

## Authors' contributions

TK designed the experiments, collected data and wrote the manuscript. KS managed the laboratory and adjusted the schedule of subjects. MK participated in the design of the study and performed statistical analysis. TI and KO supervised the study. All authors read and approved the final version of the article.
